# Which factors predict the improvement perception after a Single-Session Intervention on frontline professionals during a crisis situation?

**DOI:** 10.1192/j.eurpsy.2025.1994

**Published:** 2025-08-26

**Authors:** A. L. D. S. Ache, B. B. Montezano, B. P. Mosqueiro, M. A. Caldieraro, L. Spanemberg, G. A. Salum, M. P. Fleck

**Affiliations:** 1 Psychiatry, Hospital de Clinicas de Porto Alegre; 2 Psychiatry, Programa de Pos-Graduacao em Psiquiatria e Ciencias do Comportamento; 3 Escola de Medicina, Programa de Pos-Graduacao em Medicina e Ciencias da Saude, Escola de Medicina, Pontifıcia Universidade Catolica do Rio Grande do Sul; 4 Escola de Direito, Programa de Pos-Graduacao em Ciencias Criminais, Escola de Direito, Pontifcia Universidade Catolica do Rio Grande do Sul, Porto Alegre, Brazil

## Abstract

**Introduction:**

The COVID-19 pandemic set up a global health crisis, with an impact on the daily lives of the population. The effectiveness and importance of Single-Session Interventions (SSI), have increasingly been demonstrated, which also found expression during the Pandemic and remote care and has shown a significant impact in reducing symptoms, reducing relapses and hospitalizations, increasing positive attitudes, improving treatment adherence, and reducing the length of hospital stay for various mental disorders. It is considered an easy-to-access and economical tool.

The Hospital de Clínicas de Porto Alegre (HCPA) and the Brazilian Ministry of Health developed the TELEPSI. This Project aims to: provide telecare using different types of psychotherapeutic approaches to health professionals, teachers, and essential service workers during the Pandemic in Brazil.

**Objectives:**

This study aims to investigate the predictors of perceived improvement after the Single Session Intervention with Enhanced Psychoeducation (SSI-EP) with support videos in frontline professionals during the Covid 19 Pandemic.

**Methods:**

he COVID-19 pandemic set up a global health crisis, with an impact on the daily lives of the population. This study analyzed data from a large trial including frontline workers from April 2020 to December 2021. We included all participants randomized to SSI-EP.

**Results:**

The final sample consisted of 709 subjects, 82.8% were health professionals, 87.8% health professionals. One month after the intervention, 558 (78.7%) have improved emotional symptoms. Factors associated with better outcomes were the number of videos watched by the participants and use of medications without a medical prescription. Excessive consumption of carbohydrates and fats was negatively associated with improvement.

**Image:**

**Figure:**
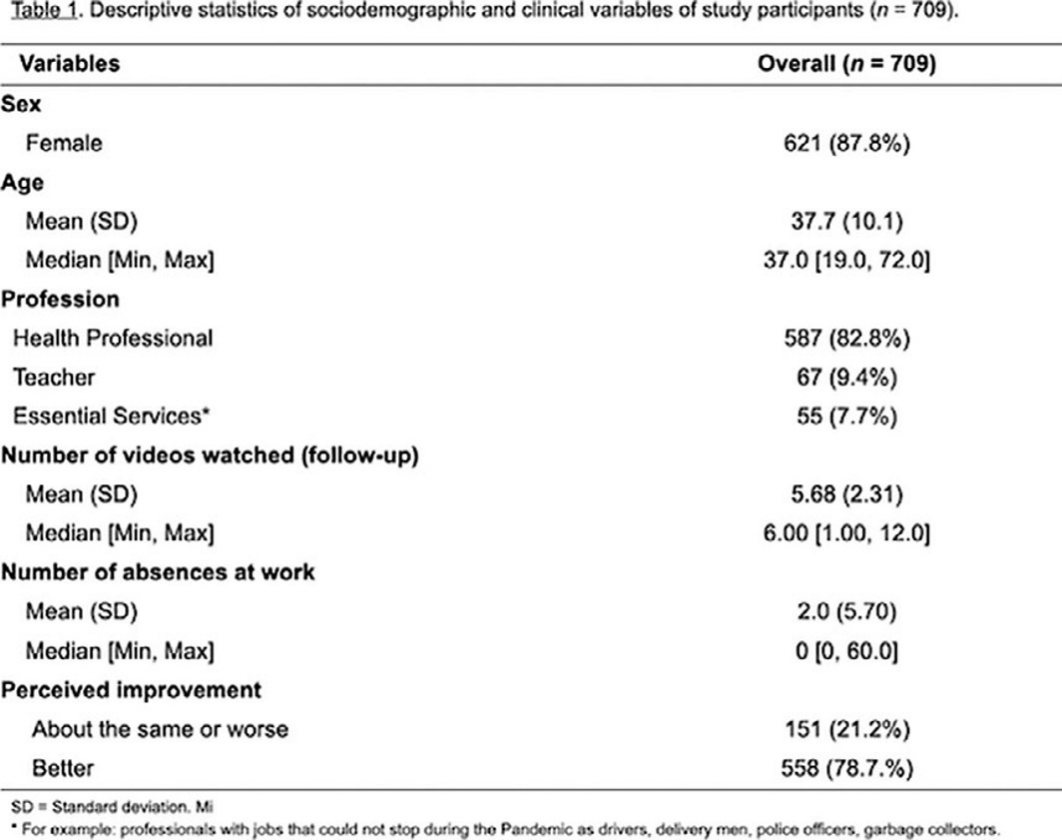

**Image 2:**

**Figure:**
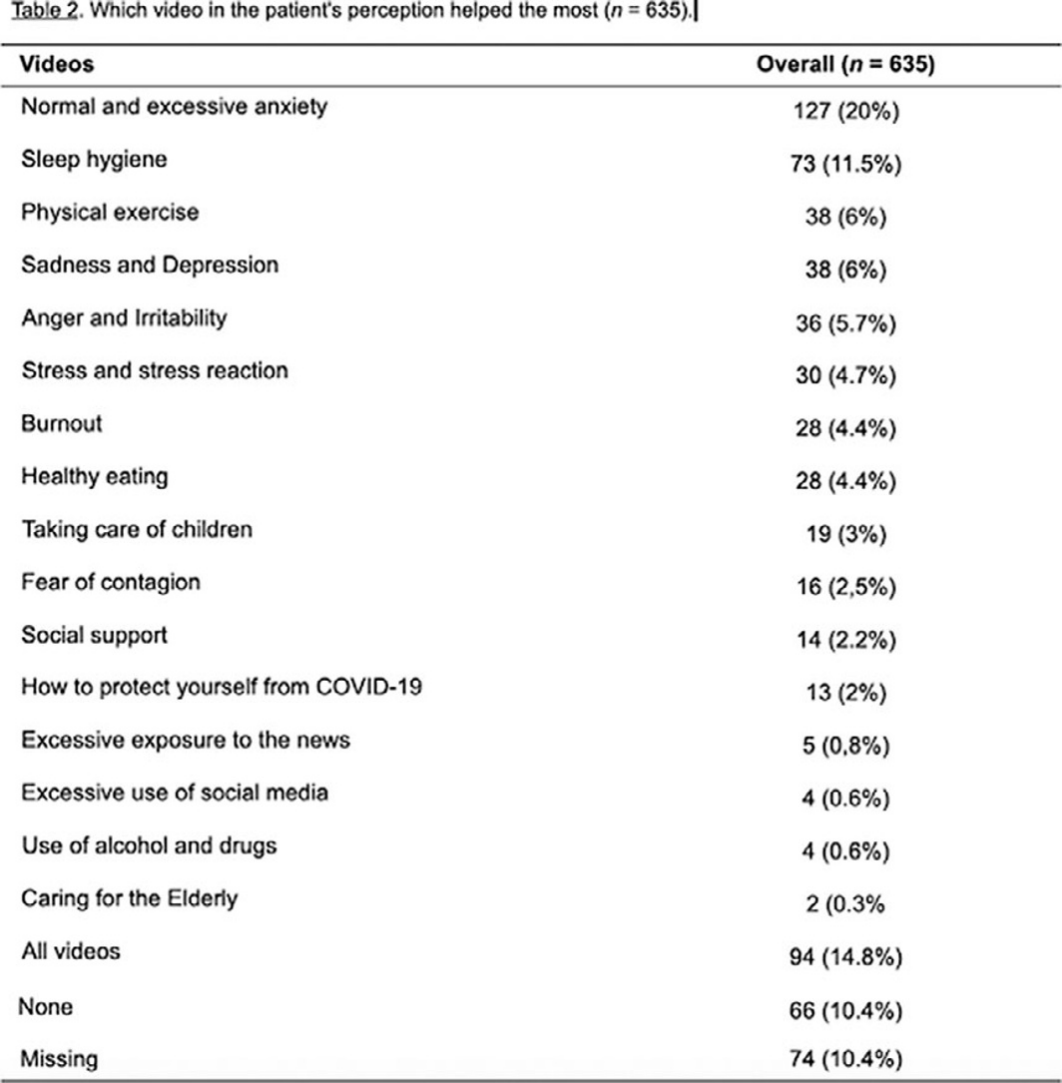

**Image 3:**

**Figure:**
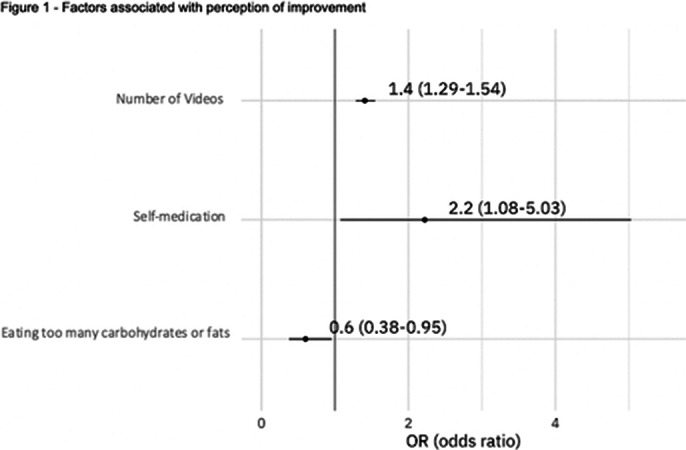

**Conclusions:**

This new TELEPSI proposal that combines online SSI based on Psychoeducation with the sending of support videos proved to be effective, as we saw in the main article in which a significant proportion of participants improved. The participant’s perception of improvement, corroborates the data found on the improvement of symptom scales and some factors associated with this outcome, such as videos. This was an interesting finding and the importance of studying and exploring and studying more and more digital interventions, which can offer access to many people and have low implementation costs. We believe that in this way we will be able to better indicate such new psychotherapeutic strategies in a more personalized and adapted way. Enhanced psychoeducation is an effective and low-cost approach to improving symptoms. More research is needed to compare its effectiveness with other intervention strategies. Furthermore, its application must be explored beyond the context of the pandemic and social isolation.

**Disclosure of Interest:**

None Declared

